# Detection of inflammatory cell function using ^13^C magnetic resonance spectroscopy of hyperpolarized [6-^13^C]-arginine

**DOI:** 10.1038/srep31397

**Published:** 2016-08-10

**Authors:** Chloé Najac, Myriam M. Chaumeil, Gary Kohanbash, Caroline Guglielmetti, Jeremy W. Gordon, Hideho Okada, Sabrina M. Ronen

**Affiliations:** 1 Department of Radiology and Biomedical Imaging, University of California San Francisco, San Francisco, CA, USA; 2 Department of Neurological Surgery, University of Surgery, University of California San Francisco, San Francisco, CA, USA; 3 Bio-imaging Lab, University of Antwerp, Antwerpen, Belgium

## Abstract

Myeloid-derived suppressor cells (MDSCs) are highly prevalent inflammatory cells that play a key role in tumor development and are considered therapeutic targets. MDSCs promote tumor growth by blocking T-cell-mediated anti-tumoral immune response through depletion of arginine that is essential for T-cell proliferation. To deplete arginine, MDSCs express high levels of arginase, which catalyzes the breakdown of arginine into urea and ornithine. Here, we developed a new hyperpolarized ^13^C probe, [6-^13^C]-arginine, to image arginase activity. We show that [6-^13^C]-arginine can be hyperpolarized, and hyperpolarized [^13^C]-urea production from [6-^13^C]-arginine is linearly correlated with arginase concentration *in vitro*. Furthermore we show that we can detect a statistically significant increase in hyperpolarized [^13^C]-urea production in MDSCs when compared to control bone marrow cells. This increase was associated with an increase in intracellular arginase concentration detected using a spectrophotometric assay. Hyperpolarized [6-^13^C]-arginine could therefore serve to image tumoral MDSC function and more broadly M2-like macrophages.

Inflammation has now been recognized as an important hallmark of cancer^1^,^2^. Inflammatory cells, and in particular myeloid-derived suppressor cells (MDSCs), promote tumor development, angiogenesis and metastasis^3^,^4^. MDSCs are a heterogeneous population of bone marrow-derived immature myeloid cells (IMCs). In healthy individuals, IMCs consist of myeloid progenitors and precursors of macrophages, dendritic cells and granulocytes^5^,^6^. In cancer, soluble cytokines such as granulocyte macrophage colony stimulating factor (GM-CSF), granulocyte colony stimulating factor (G-CSF), interleukin-13 (IL-13), interleukin-4 and interferon-γ are secreted into the tumor microenvironment. These factors induce a partial differentiation of IMCs, which results in the proliferation and activation of MDSCs^6^,^7^. Myeloid cells make up to ~40% of the tumor mass in several cancers, and recent studies show up to a ten-fold increase in MDSCs in the peripheral blood, and a correlation between high levels of MDSCs and tumor burden^6^,^8^,^9^,^10^,^11^,^12^,^13^,^14^,^15^,^16^.

The pro-tumorigenic role of MDSCs is mediated by their ability to suppress several aspects of the host immune response, and most notably by their ability to inhibit T-cell proliferation and function^5^,^11^. Immunotherapeutic strategies that target MDSCs and block their expansion and activation are therefore emerging^17^,^18^,^19^,^20^. MDSCs act via different mechanisms, including production of high levels of reactive oxygen species, induction of regulatory T-cells, and depletion of cysteine. Additionally, similarly to M2-type macrophages^21^,^22^, MDSCs up-regulate the expression of arginase (ARG)^11^,^23^,^24^,^25^, and a subset of MDSCs, the mononuclear MDSCs that represent ~30% of the population, also expresses elevated levels of the enzyme inducible nitric oxide synthase (iNOS)^3^,^26^. Both ARG and iNOS deplete L-arginine from the tumor microenvironment by catalyzing the hydrolysis of L-arginine into L-ornithine and urea, or into L-citrulline and nitric oxide, respectively^6^. Amongst the aforementioned factors secreted by the tumor, IL-13 is one of the key factors inducing ARG activity in MDSCs^7^,^27^. Because T-cells require arginine for their proliferation, the decreased level of L-arginine plays a central role in T-cell inhibition. Probing arginine metabolism could therefore serve as a readout of MDSC activity and as a method to detect the inhibition of this activity in response to MDSC-targeted treatment.

Magnetic resonance imaging (MRI) approaches that specifically image MDSCs and their response to immunotherapy have been limited. One study, performed in a model of murine breast carcinoma, detected MDSCs *in vivo* using perfluorocarbon (PFC)-based ^19^F MRI^28^. However, this approach is unable to probe cell function. ^13^C magnetic resonance spectroscopy (^13^C-MRS) and spectroscopic imaging (MRSI) inform on real-time metabolic fluxes by probing conversion of exogenous ^13^C-labeled substrates. Dissolution dynamic nuclear polarization (DNP) offers the unique ability to hyperpolarize and dissolve ^13^C-labeled compounds in solution, enabling more than 10,000-fold enhancement in the signal to noise ratio (SNR) of labeled substrates and their metabolic products compared to thermally polarized compounds^29^.

To achieve the improved SNR, hyperpolarization requires that the ^13^C-labeled compound be mixed with a free radical and placed at low temperature (<2K) and at high magnetic field (~3–5T). Microwave irradiation then saturates the electron spin resonance and polarization is transferred from the radical electron to the labeled nucleus^29^,^30^. Hyperpolarized agents are characterized by their polarization enhancement, which represents the efficiency of the DNP method at increasing the SNR. Hyperpolarized agents are also characterized by their lifetime, or the longitudinal T_1_ relaxation time of the polarized carbon, which determines how fast the polarization is lost after dissolution. Hyperpolarized lifetimes depend on the chemical structure and labeling position of the compound, and are typically less than a minute^29^,^30^. In the case of carbonyl-labeled probes, which are the most commonly labeled, the T_1_ is dominated by chemical shift anisotropy (CSA) and therefore benefits from lower magnetic field strengths such as those used in the clinic (1.5–3 Tesla) and for which the CSA is reduced (CSA*-*α B_0_^2^) and the *T*_1_ is longer^31^,^32^,^33^,^34^,^35^.

Over the past decade, several hyperpolarized ^13^C probes have been developed and applied to the imaging of normal and diseased tissue^30^,^36^. The most common probe, hyperpolarized [1-^13^C]-pyruvate, has been widely used in cell and animal models of cancer, wherein elevated production of hyperpolarized [1-^13^C]-lactate can serve to detect the presence of tumor cells, and a drop in hyperpolarized [1-^13^C]-lactate is associated with response to treatment^30^,^37^,^38^,^39^,^40^. Hyperpolarized [1-^13^C]-pyruvate has also been applied to the study of other diseases^41^,^42^,^43^,^44^,^45^,^46^,^47^. In addition, the first-in-human study performed on prostate cancer patients at the University of California, San Francisco, confirmed the potential of this imaging method in the clinic^48^. Interestingly, recent studies have also shown that elevated levels of hyperpolarized [1-^13^C]-lactate correlate with inflammation in lung injury and arthritis^45^,^49^, demonstrating the value of hyperpolarized [1-^13^C]-pyruvate for imaging the presence of inflammatory cells. However, lactate up-regulation from inflammatory cells and tumor cells are indistinguishable, limiting the utility of hyperpolarized [1-^13^C]-pyruvate as a specific probe for the detection of MDSCs in cancer.

Considering the role of ARG in MDSC function, we instead focused on using hyperpolarized [6-^13^C]-arginine as a probe to investigate ARG activity. We first characterized this new hyperpolarized probe and then show that hyperpolarized [^13^C]-urea production from hyperpolarized [6-^13^C]-arginine linearly correlates with *in vitro* ARG enzyme activity. Furthermore, we demonstrate that we can detect hyperpolarized [^13^C]-urea production from hyperpolarized [6-^13^C]-arginine in activated MDSCs but not in control bone marrow (BM) cells, confirming the utility of hyperpolarized [6-^13^C]-arginine as a probe for monitoring ARG expression in cells.

## Results

### Characterization of hyperpolarized [6-^13^C]-arginine

**Figure 1:**
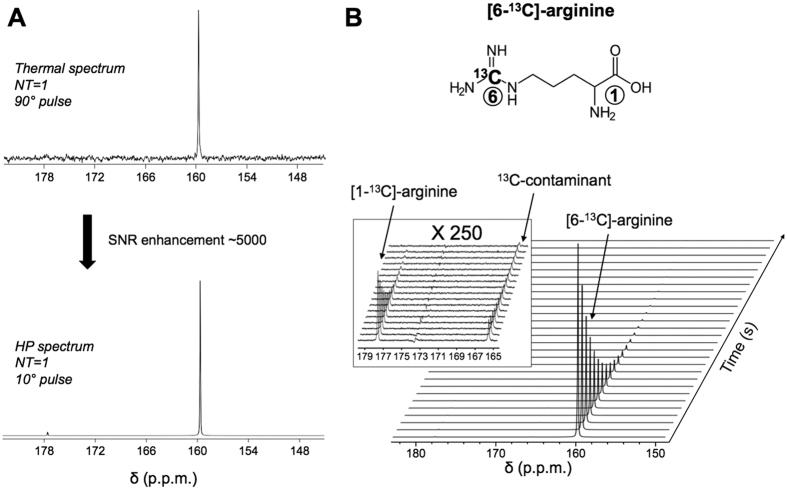
[6-^13^C]-arginine can be hyperpolarized. (**A**) [6-^13^C]-arginine thermal equilibrium spectrum (top) and hyperpolarized spectrum (bottom) acquired at 11.7 Tesla showing the ~5000-fold SNR enhancement by the dissolution dynamic nuclear polarization technique (NT = number of transient). (**B**) Stack plot of ^13^C MR spectra of hyperpolarized [6-^13^C]-arginine in solution acquired at 11.7 Tesla showing decay of the hyperpolarized signals (temporal resolution 3 s). Resonances of [6-^13^C]-arginine (δ_[6-13C]-arginine_ = 159.7 ppm), [1-^13^C]-arginine (δ_[1-13C]-arginine_ = 177.1 ppm, originating from 1.1% ^13^C natural abundance at C1 position), ^13^C-contaminant (at the same resonance of urea, δ_[13C]-urea_ = 165.5 ppm, and that could originate from 1.1% ^13^C natural abundance of urea contaminant) were detectable.

To validate the hypothesis that hyperpolarized [6-^13^C]-arginine can serve as an imaging probe for MDSC activity and function, we first determined the enhancement in polarization that can be achieved for this new probe, and its longitudinal relaxation time T_1_. Following dissolution, the resonance of [6-^13^C]-arginine was detected (δ_[6-13C]-arginine_ = 159.7 ppm) and a polarization enhancement of 5018 ± 412 fold was observed at 37 °C at 11.7 Tesla when compared to the thermal equilibrium spectrum (Fig. 1A). The resonance of [1-^13^C]-arginine (δ_[1-13C]-arginine_ = 177.1 ppm, originating from 1.1% ^13^C natural abundance at the C1 position) was also detected. Additionally, at 11.7 Tesla, a resonance at 165.5 ppm, which corresponds to the resonance of [^13^C]-urea, was also observed and could originate from 1.1% ^13^C natural abundance of a urea contaminant (Fig. 1B). The T_1_ values of all detected resonances were measured in solution at 11.7 and at 3 Tesla and are reported in Table 1. The data show that the T_1_ of hyperpolarized [6-^13^C]-arginine was comparable at 3 Tesla and 11.7 Tesla (9.9 ± 0.1 s at 11.7 Tesla and 12.3 ± 0.8 s at 3 Tesla).

### Hyperpolarized [6-^13^C]-arginine as an imaging probe for arginine metabolism

**Figure 2:**
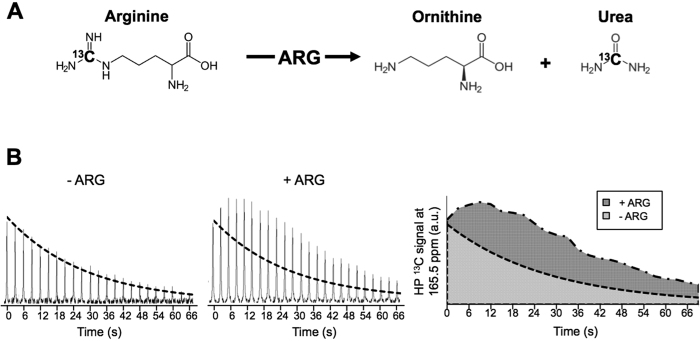
[^13^C]-urea detection: metabolic conversion and post-processing analysis. (**A**) Schematic showing the conversion of hyperpolarized [6-^13^C]-arginine into hyperpolarized [^13^C]-urea and ornithine by ARG. The ^13^C labeled carbon is highlighted in bold. (**B**) Mono-exponential decay (doted curve) depending on measured hyperpolarized ^13^C-contaminant T_1_ and flip angle was subtracted from all data allowing detection of urea production when ARG is present.

Next it was necessary to confirm that conversion of arginine into its metabolic products can be detected (Fig. 2A). To this end, different concentrations of ARG (0, 300, 667, 1334, 2000 U/L), the enzyme that catalyzes the conversion of arginine into urea and ornithine, were exposed to hyperpolarized [6-^13^C]-arginine, and dynamic ^13^C spectra were acquired every 3 seconds.

To correct for the contaminant signal detected at 165.5 ppm in the arginine solution, a mono-exponential decay curve depending on the flip angle and on the hyperpolarized ^13^C-contaminant T_1_ measured previously in solution (24.6 ± 1.9 s at 11.7 Tesla) was subtracted from all hyperpolarized ^13^C dynamic datasets. As illustrated in Fig. 2B, this post-processing operation separates the contaminant from signal originating from conversion of hyperpolarized [6-^13^C]-arginine into [^13^C]-urea.

**Figure 3:**
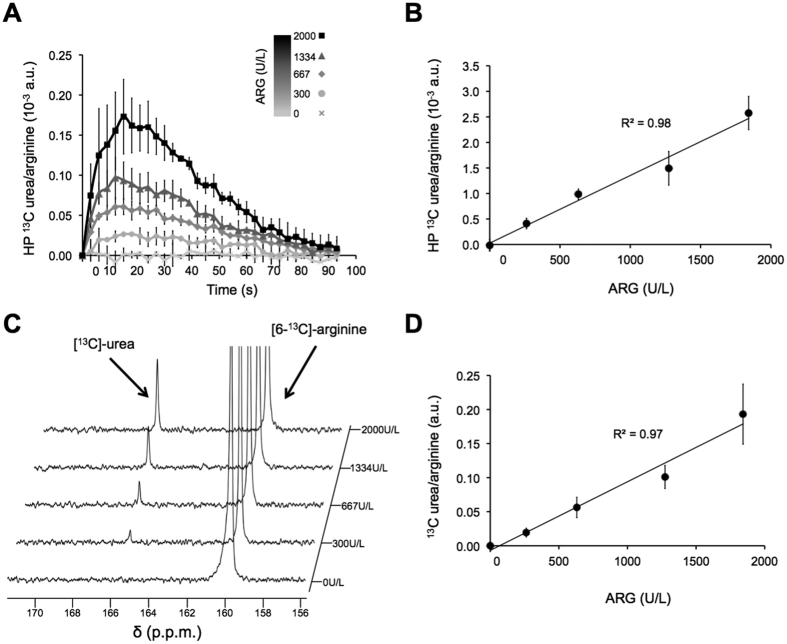
Hyperpolarized [^13^C]-urea production is linearly correlated to ARG concentration. (**A**) Build-up of hyperpolarized [^13^C]-urea normalized to area-under-the-curve of hyperpolarized [6-^13^C]-arginine over time illustrating increased urea production with ARG concentration. Maximum at 16.5 ± 4.5 s post maximum arginine signal was observed with ARG concentration at and above 300 U/L. (**B**) Area-under-the-curve (AUC) of hyperpolarized [^13^C]-urea build-up as a function of ARG concentration, showing a correlation between hyperpolarized [^13^C]-urea production and enzyme concentration. (**C**) Stack-plot of thermal equilibrium spectra and (**D**) [^13^C]-urea and [6-^13^C]-arginine integral ratios from thermal equilibrium spectra as a function of ARG concentration, confirming hyperpolarized [^13^C]-urea detection.

After correction for the contaminant, a detectable build-up of hyperpolarized [^13^C]-urea at 165.5 ppm with a maximum at 16.5 ± 4.5 s post maximum arginine signal was observed when ARG concentration was at or above 300 U/L (Fig. 3A). Furthermore, the hyperpolarized [^13^C]-urea to [6-^13^C]-arginine area-under-the-curve (AUC) ratio increased linearly with enzyme concentration, consistent with increased urea production with increased enzyme concentration (R^2^ = 0.98, Fig. 3B). Continued production of urea was confirmed in thermal equilibrium ^13^C spectra acquired after the end of the hyperpolarized study. These data also showed a linear increase of the [^13^C]-urea to [6-^13^C]-arginine peak integral ratios with enzyme concentration (R^2^ = 0.97, Fig. 3C,D).

### Studies of MDSCs

**Figure 4:**
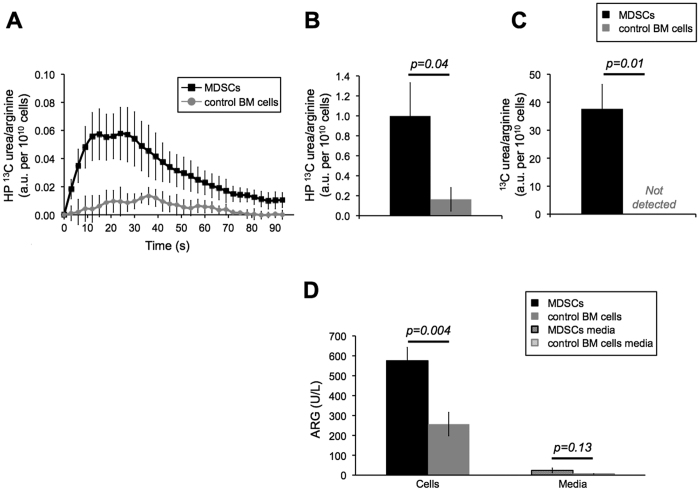
Hyperpolarized [^13^C]-urea production is detected in MDSCs. (**A**) Build-up of hyperpolarized [^13^C]-urea normalized to area-under-the-curve of hyperpolarized [6-^13^C]-arginine as a function of time and (**B**) hyperpolarized [^13^C]-urea/[6-^13^C]-arginine area-under-the-curve ratio for MDSCs and control BM cells showing higher hyperpolarized [^13^C]-urea in MDSCs compared to control BM cells. (**C**) Ratio of [^13^C]-urea and [6-^13^C]-arginine integrals from thermal equilibrium spectra showing detection of [^13^C]-urea only in MDSCs and (**D**) ARG enzyme concentration measured by spectrophotometric assay for MDSCs, control BM cells and growth media exposed to cells consistent with the hyperpolarized results.

We next examined arginine metabolism using ^13^C MRS and hyperpolarized [6-^13^C]-arginine in MDSCs generated by culturing bone marrow cells with IL-13 and in control BM cells cultured without IL-13. As illustrated in Fig. 4A, injection of hyperpolarized [6-^13^C]-arginine into an NMR tube containing MDSCs resulted in a clearly detectable build-up of hyperpolarized [^13^C]-urea and, importantly, this build-up was significantly higher than that observed in control BM cells. The ratio of hyperpolarized [^13^C]-urea AUC to [6-^13^C]-arginine AUC in MDSCs was significantly higher than that observed in control BM cells (1.0 ± 0.3 a.u. per 10^10^ cells in MDSCs (n = 3) versus 0.2 ± 0.1 a.u. per 10^10^ cells control BM cells (n = 4), *p-value* = 0.04, Fig. 4B). Production of urea was also confirmed in MDSCs with thermal equilibrium ^13^C spectra, but was below detection in control BM cells resulting in a significant increase in the ratio of [^13^C]-urea to [6-^13^C]-arginine (*p-value* = 0.01, n = 3 MDSCs, n = 4 control BM cells, Fig. 4C). No hyperpolarized [^13^C]-urea build-up was detected in the growth media that had been exposed to either MDSCs or control BM cells. No [6-^13^C]-citrulline production, potentially mediated by iNOS, could be detected. These results indicated that ^13^C MRS and hyperpolarized [6-^13^C]-arginine could detect an increase in intracellular ARG concentration following IL13 treatment and activation of MDSCs.

To further confirm that IL13 treatment had effectively converted BM cells into MDSCs and up-regulated ARG production, and that the concentration of ARG in the growth medium was below detection by our hyperpolarized method, spectrophotometric assays were performed on MDSCs, control BM cells, and samples of growth media exposed to cells. Assays confirmed a significant increase in intracellular ARG in activated MDSCs (576 ± 67 U/L in MDSCs versus 256 ± 59 U/L in control BM cells, *p-value* = 0.004, Fig. 4D). Extracellular ARG concentrations were much lower and well below the ~300 U/L level shown to be detectable in our enzyme studies. More ARG was observed in the extracellular medium of MDSCs compared to control BM cells, but the difference did not reach statistical significance (7 ± 1 U/L in control BM cells medium versus 24 ± 12 U/L in MDSC medium, *p-value* = 0.13, Fig. 4D). These results showed that the hyperpolarized method was able to detect the up-regulation of ARG expression that occurs in activated MDSCs.

## Discussion

The goal of our study was to assess the value of hyperpolarized arginine as a probe to monitor ARG activity and, as such, to develop a novel method for detection of active MDSCs, which increase their expression of ARG to mediate T-cell inhibition and cancer immune evasion. To this end we used arginine labeled on the guanidino group [6-^13^C]. Hyperpolarized agents are most commonly labeled on the carbonyl group^31^,^32^,^33^,^34^,^35^. However, here, labeling of arginine on the carbonyl group would result in a chemical shift difference of only 0.2 ppm between the resonances of arginine (δ_[1-13C]-arginine_ = 177.1 ppm) and its ARG-mediated metabolic product ornithine (δ_[13C]-ornithine_ = 176.9 ppm) or its iNOS-mediated metabolic product citrulline (δ_[1-13C]-citrulline_ = 177.3 ppm). Labeling of the carbonyl group would therefore not provide the necessary spectral resolution to separate substrate and products. Similar considerations would hold for any of the protonated carbons of the arginine molecule, in addition to very short T_1_ values due to the presence of attached protons, as previously discussed^30^,^36^,^50^. In contrast, labeling the guanidino position provides adequate separation between the resonances of arginine (δ_[6-13C]-arginine_ = 159.7 ppm) and its ARG-mediated metabolic product urea (δ_[13C]-urea_ = 165.5 ppm). Labeling of the guanidino group could also enable detection of iNOS activity, the enzyme up-regulated by mononuclear MDSCs and that would lead to the conversion of hyperpolarized arginine into citrulline (δ_[6-13C]-citrulline_ = 164.2 ppm).

We showed that [6-^13^C]-arginine could be successfully polarized. An SNR enhancement of ~5000 fold at the time of acquisition was observed, allowing rapid detection of our hyperpolarized ^13^C-labeled probe, the ^13^C natural abundance of the carbonyl, and the production of urea by ARG. The longitudinal relaxation time T_1_ of this new probe was found to be relatively short (T_1_ = 9.9 ± 0.1 s at 11.7 Tesla) as compared to other probes and most notably the extensively used pyruvate probe (~48 s at 11.7 Tesla)^30^,^36^,^50^. The T_1_ of the guanidino carbon is probably strongly affected by the quadrupolar relaxation that results from the strong scalar interaction with the three surrounding ^14^N atoms. In urea, this relaxation mechanism was shown to lead to a strong decrease in T_1_ during sample transfer through low field between the polarizer and the MR scanner^51^. A similar phenomenon is likely occurring here. Importantly, because the magnitude of this effect could not be easily estimated, it was not possible for us to back-calculate the polarization level to the time of dissolution (and prior to transfer through low field), as has been reported for other probes^30^. The T_1_ of our guanidino-labeled probe also showed little dependence on magnetic field (T_1_ = 9.9 ± 0.1 s and 12.3 ± 0.8 at 11.7 and 3 Tesla respectively in solution at 37°). This is in contrast to several other probes labeled at the carbonyl carbon for which the T_1_ is longer at lower field strengths, dihydroascorbate (T_1_~56 s at 3 Tesla and ~21 s at 11.7 Tesla), alanine (T_1_~42 s at 3 Tesla and ~29 s at 9.4 Tesla), alpha-ketoglutarate (T_1_~52 s at 3 Tesla and ~19 s at 11.7 Tesla)^32^,^33^,^34^,^35^ or [1-^13^C]-arginine (T_1_ = 24.8 ± 3.9 s at 3 Tesla and 13.4 ± 0.9 s at 11.7 Tesla as determined in this study).

Our studies investigated BM cells treated with the stimulating factors GM-CSF, G-CSF, and IL-13 previously reported to accurately model MDSCs and control BM cells^7^,^27^,^52^. Following *in vitro* injection of hyperpolarized [6-^13^C]-arginine, we measured the urea-to-arginine AUC ratio. This provided us with a simple, assumption-free and model-free method to study the ARG reaction^53^. We found that a build-up of hyperpolarized [^13^C]-urea was observed in IL13-treated MDSCs, but not in control BM cells, in line with the previously reported enhanced expression of ARG in MDSCs as compared to controls^7^,^27^,^52^. However, we were not able to detect production of [^13^C]-urea in the extracellular medium that had been exposed to MDSCs. MDSCs can deplete the arginine pool that is required for T-cell activity by secreting arginase into the extracellular space and/or by taking up arginine and breaking it down within the cell^54^,^55^. A recent study in murine MDSCs showed an increase in CAT-2B, the arginine transporter^54^. Accordingly, and consistent with our findings, we would expect a significant amount of arginine to be rapidly taken up and metabolized by intracellular ARG in our murine MDSCs. Importantly, ARG present in the intracellular compartment would remain concentrated. In contrast, any ARG released from our MDSCs into the large volume of cell culture medium would be greatly diluted, and thus its concentration could be below detection using hyperpolarized [6-^13^C]-arginine, as indicated by our studies.

When considering studies in humans, it is important to note that human MDSCs do not overexpress CAT-2B, suggesting that the majority of ARG is released from human MDSCs into the extracellular space resulting in elevated levels of ARG within the tumor microenvironment^54^. This would likely accelerate hyperpolarized [6-^13^C]-arginine metabolism and potentially help in the *in vivo* clinical detection of hyperpolarized urea production by ARG in patients. Nonetheless, prior to *in vivo* translation, approaches to increase the T_1_ of hyperpolarized [6-^13^C]-arginine and to enhance the detection of [^13^C]-urea should be considered. For example, to increase the T_1_, [6-^13^C, ^15^N_3_]-arginine could be used. This would eliminate the quadrupolar relaxation, although splitting as a result of the ^13^C-^15^N J-coupling (J(CN) for the guanidino group ~20 Hz 56) could increase the complexity of the spectrum and limit the improvement in SNR. Transporting of hyperpolarized arginine in a magnetic carrier, which has been shown to decrease the quadrupolar relaxation effect for hyperpolarized [^13^C]-urea, could also be considered^57^. Another approach would consist in extending the T_1_ through deuteration as previously demonstrated in other molecules^58^. Additionally, the dose of arginine injected *in vivo* should be maximized. Fortunately, arginine is a semi-essential amino acid commonly used as a supplement, and a recent study recommended arginine intake up to 20 g per day^59^. Injection of elevated concentrations of hyperpolarized [6-^13^C]-arginine can therefore be safely considered and could result in rapid production of detectable levels of hyperpolarized [^13^C]-urea in the tumor microenvironment. Optimized pulse sequences could also be implemented to increase the SNR of [^13^C]-urea. For instance, a multiband pulse sequence that applies a small flip angle to [6-^13^C]-arginine to preserve its magnetization while a larger flip angle is applied to [^13^C]-urea to increase its SNR, would improve the likelihood of detecting metabolism^60^. The use of an automated pump for rapid injection of hyperpolarized [6-^13^C]-arginine after dissolution with limited transfer through low field^61^, could also improve the likelihood of detecting ARG activity. Finally, polarization levels could be increased using the SpinLab clinical polarizer^62^,^63^, which has a higher field strength and lower temperature, and has been shown to enhance the polarization level of other agents^64^.

In conclusion, we report here, for the first time, the use of a hyperpolarized ^13^C MRS probe to specifically monitor the function of MDSCs *in vitro*. MDSCs play a major role in cancer by promoting tumor immune evasion through inhibition of T-cell proliferation and anti-tumoral activity^1^,^5^,^65^,^66^. Additional optimization approaches are required before *in vivo* translation of this probe. Nonetheless, if successful, this probe could provide a novel non-invasive imaging method for monitoring MDSC activity to inform on the role of MDSCs in tumor development and their inhibition by emerging MDSC-targeted immunotherapies^17^,^18^,^19^,^20^. This probe could also be useful in inflammatory diseases to monitor the modulation of macrophage phenotype in response to anti-inflammatory therapies^21^,^22^,^67^.

## Material and Methods

### [6-^13^C]-Arginine Hyperpolarization

[6-^13^C]-arginine (Cambridge Isotopes Laboratories, USA) was dissolved to a concentration of 3.4 M in water containing 7.5 μM of Trizma^®^ base (Sigma-Aldrich, USA). The mixture was heated to 50 °C, sonicated and vortexed until the content was fully dissolved. 15 mM of trityl radical OX063 (Oxford Instruments, UK) and 1.5 mM Gadolinium-Dotarem (Macrocyclics, USA) were then added. For all experiments, aliquots (~72 mg) were polarized using a HyperSense DNP system (Oxford Instruments) for ~75 minutes (3.35 Tesla, 1.4 K, 94.067 GHz) and subsequently rapidly dissolved in a Tris-based buffer (40 mM Tris, 3 μM Na_2_EDTA, pH ~7.8) to yield ~50 mM solutions of hyperpolarized [6-^13^C]-arginine as previously described for other probes^29^,^30^.

### Relaxation time and polarization levels

Following dissolution, hyperpolarized [6-^13^C]-arginine was placed either in a 10 mm NMR tube (number of repeats (n) = 3, 11.7 Tesla INOVA spectrometer, Agilent Technologies, USA) or in a 5 mL syringe (n = 2, 3 Tesla clinical scanner, GE Healthcare, USA). Dynamic ^13^C spectra were acquired using a non-localized single pulse (parameters at 11.7 Tesla: TR = 3 s, flip angle (FA) = 10 degree, number of transients (NT) = 50, spectra width (SW) = 20 kHz, 20000 points, 10 mm broadband probe; parameters at 3 Tesla: TR = 2 s, FA = 15 degree, NT = 60, SW = 5 kHz, 2048 points, dual ^1^H/ ^13^C volume coil). T_1_ of hyperpolarized [6-^13^C]-arginine (δ_[6-13C]-arginine_ = 159.7 ppm), [1-^13^C]-arginine (δ_[1-13C]-arginine_ = 177.1 ppm) and ^13^C-contaminant present in the mixture (δ_13C-contaminant_ = 165.5 ppm) were determined by quantifying the area under the peak, correcting for flip angle, and fitting the signal decay curve to a mono-exponential. Following total decay of the hyperpolarized signal, a thermal equilibrium spectrum was acquired at 11.7 Tesla using FA = 90 degree, TR = 80 s, NT = 16 and other acquisition parameters identical to the ones mentioned above (n = 3). The level of polarization in solution was calculated by comparing the signal on the first hyperpolarized spectrum of the dynamic data set to the corresponding signal in the thermal equilibrium spectrum after correction for flip angle and number of transients.

### *In vitro* enzyme experiments using hyperpolarized ^13^C MR

Arginase enzyme (Abcam, UK) was dissolved in 500 μL of Tris-based dissolution buffer at different concentrations (0, 300, 667, 1334, 2000 U/L, n = 3 per enzyme concentration) and placed in a 10 mm NMR tube at 37 °C. Within ~18 seconds following dissolution, 3 mL of hyperpolarized [6-^13^C]-arginine was injected into the NMR tube. Immediately after injection, dynamic ^13^C spectra were acquired on the 11.7 Tesla INOVA spectrometer (Agilent Technologies, USA) using a 10 mm broadband probe (TR = 3 s, FA = 10 degree, NT = 50, SW = 20 kHz, 20000 points), followed by thermal equilibrium spectrum after complete decay of the hyperpolarized signal (TR = 80 s, FA = 90 degree, NT = 16, SW = 20 kHz, 20000 points).

### Cell model: MDSC generation from bone marrow cells

All animal research was approved by the Institutional Animal Care and Use Committee of the University of California, San Francisco. All experiments were performed in accordance with relevant guidelines and regulations. MDSCs were generated essentially as previously described^7^,^27^,^52^. Briefly, Balb/c mice (n = 10 per group and per experiment, 6 weeks old; The Jackson Laboratory, USA) were used in this study. Red blood cell-depleted BM cells were isolated and cultured in 6-well plates under standard conditions (37 °C humidified atmosphere at 5% CO_2_ and 95% air) in high-glucose DMEM (Mediatech Inc., USA) supplemented with 10% heat-inactivated fetal bovine serum (Mediatech Inc., USA), 100 U/mL penicillin and 100 mg/mL streptomycin (UCSF Cell Culture Facility). Cells were separated into two groups (10 plates per group): control BM cells and MDSCs. On days 0, 4 and 9, granulocyte colony-stimulating factor (G-CSF, 0.1 μg/mL, Shenandoah Biotechnology Inc., USA) and granulocyte macrophage colony-stimulating factor (GM-CSF, 250 U/mL, R&D Systems, USA) were added to both cell groups culture media. On day 4 and 9, IL13 (80 ng/mL, Preprotech, USA) was added to MDSCs only. On day 10, cells and growth media were collected to perform hyperpolarized ^13^C MR studies. Cells were counted for data normalization. For each group, media and cells samples were reserved for spectrophotometric assay.

### Hyperpolarized ^13^C MR studies using cell suspensions

Cells from all the culture wells except one were collected (total of 1.9 ± 0.8 × 10^7^ MDSCs (n = 3) or control BM cells (n = 4)). For each group, (1) cells in 500 μL of their growth culture media (n = 3/4 MDSCs/control BM cells) and (2) 500 μL of growth medium exposed to cells (n = 5 MDSCs/control BM cells) were placed in a 10 mm NMR tube at 37 °C to assess the intra- and extracellular ARG activity respectively. Within ~18 seconds following dissolution, 3 mL of hyperpolarized [6-^13^C]-arginine was injected into the NMR tube. Dynamic and thermal equilibrium ^13^C spectra were then acquired as described above for the hyperpolarized ^13^C MR enzyme studies.

### MR data analysis

Following phase correction and baseline subtraction, spectra were quantified by peak integration using MestRenova (Mestrelab Research S.L., Spain). For dynamic acquisitions, [^13^C]-urea integrals were then normalized to the area-under-the-curve (AUC) of hyperpolarized [6-^13^C]-arginine. A mono-exponential decay curve that depends both on hyperpolarized ^13^C-contaminant T_1_ and flip angle was then subtracted from hyperpolarized [^13^C]-urea signal decay to correct for the presence of the contaminant. The area underneath the normalized hyperpolarized [^13^C]-urea build-up curve (i.e. ratio of hyperpolarized [^13^C]-urea AUC and [6-^13^C]-arginine AUC) was then quantified. For thermal equilibrium acquisitions, the ratio of [^13^C]-urea and [6-^13^C]-arginine integrals was measured.

### Spectrophotometric enzyme assay

At day 10 MDSCs/control BM cells (5.8 ± 2.3 × 10^5^ cells, n = 3 per group) from one culture well not used for hyperpolarized studies were lysed in 2 μL of 10 mM Tris-HCl buffer containing 0.4% (w/v) Triton X-100 (Sigma-Aldrich, USA) and 0.5 μL/mL protease inhibitor cocktail (Calbiochem, USA). Lysates were centrifuged at 14,000 r.p.m. for 10 min at 4 °C. ARG concentration was then measured in MDSC/control BM cell lysates and their growth media (n = 3 per group) using the QuantiChrome^TM^ arginase assay detection kit (DARG-200, BioAssays Systems, USA) following manufacturer instructions. Optical density was determined at 430 nm using an Infinite 300 m200 spectrophotometer (Tecan Systems, Inc., USA). ARG concentration in NMR tube at the time of hyperpolarized experiments was back calculated from the assays.

### Statistical analysis

All results are expressed as mean ± s.d. To determine the statistical significance of differences, an unpaired two-tailed Student’s *t*-test with unequal variance was used, with a *p*-value < 0.05 considered as significant.

## Additional Information

**How to cite this article**: Najac, C. *et al*. Detection of inflammatory cell function using ^13^C magnetic resonance spectroscopy of hyperpolarized [6-^13^C]-arginine. *Sci. Rep.* **6**, 31397; doi: 10.1038/srep31397 (2016).

## Figures and Tables

**Table 1:** *In vitro* T_1_ relaxation times measured in solution at 37 °C at 3 Tesla (n = 2) and 11.7 Tesla (n = 3).

	Relaxation time T_1_ (s)	
	3 Tesla	11.7 Tesla
[6-^13^C]-arginine	12.3 ± 0.8	9.9 ± 0.1
[1-^13^C]-arginine	24.8 ± 3.9	13.4 ± 0.9
^13^C-contaminant	*not detected*	24.6 ± 1.9

## References

[b1] Colotta F., Allavena P., Sica A., Garlanda C. & Mantovani A. Cancer-related inflammation, the seventh hallmark of cancer: links to genetic instability. Carcinogenesis 30, 1073–1081 (2009). 19468060 10.1093/carcin/bgp127

[b2] Hanahan D. & Weinberg R. A. Hallmarks of cancer: the next generation. Cell 144, 646–674 (2011). 21376230 10.1016/j.cell.2011.02.013

[b3] Condamine T., Ramachandran I., Youn J. I. & Gabrilovich D. I. Regulation of tumor metastasis by myeloid-derived suppressor cells. Annual review of medicine 66, 97–110 (2015). 10.1146/annurev-med-051013-052304 PMC4324727 25341012

[b4] Murdoch C., Muthana M., Coffelt S. B. & Lewis C. E. The role of myeloid cells in the promotion of tumour angiogenesis. Nature reviews. Cancer 8, 618–631 (2008). 18633355 10.1038/nrc2444

[b5] Talmadge J. E. & Gabrilovich D. I. History of myeloid-derived suppressor cells. Nature reviews. Cancer 13, 739–752 (2013). 10.1038/nrc3581 PMC4358792 24060865

[b6] Gabrilovich D. I. & Nagaraj S. Myeloid-derived suppressor cells as regulators of the immune system. Nature reviews. Immunology 9, 162–174 (2009). 10.1038/nri2506 PMC2828349 19197294

[b7] Kohanbash G. . GM-CSF promotes the immunosuppressive activity of glioma-infiltrating myeloid cells through interleukin-4 receptor-alpha. Cancer research 73, 6413–6423 (2013). 24030977 10.1158/0008-5472.CAN-12-4124 PMC3829000

[b8] Reardon D. A. . Immunotherapy advances for glioblastoma. Neuro-oncology 16, 1441–1458 (2014). 25190673 10.1093/neuonc/nou212 PMC4201077

[b9] Diaz-Montero C. M. . Increased circulating myeloid-derived suppressor cells correlate with clinical cancer stage, metastatic tumor burden, and doxorubicin-cyclophosphamide chemotherapy. Cancer immunology, immunotherapy: CII 58, 49–59 (2009). 18446337 10.1007/s00262-008-0523-4 PMC3401888

[b10] Ochoa A. C., Zea A. H., Hernandez C. & Rodriguez P. C. Arginase, prostaglandins, and myeloid-derived suppressor cells in renal cell carcinoma. Clinical cancer research: an official journal of the American Association for Cancer Research 13, 721s–726s (2007). 17255300 10.1158/1078-0432.CCR-06-2197

[b11] Kohanbash G. & Okada H. Myeloid-derived suppressor cells (MDSCs) in gliomas and glioma-development. Immunological investigations 41, 658–679 (2012). 23017140 10.3109/08820139.2012.689591

[b12] Thaci B. . Depletion of myeloid-derived suppressor cells during interleukin-12 immunogene therapy does not confer a survival advantage in experimental malignant glioma. Cancer gene therapy 21, 38–44 (2014). 24434573 10.1038/cgt.2013.81 PMC4035218

[b13] Chae M. . Increasing glioma-associated monocytes leads to increased intratumoral and systemic myeloid-derived suppressor cells in a murine model. Neuro-oncology 17, 978–991 (2015). 25537019 10.1093/neuonc/nou343 PMC5654350

[b14] Mirghorbani M., Van Gool S. & Rezaei N. Myeloid-derived suppressor cells in glioma. Expert review of neurotherapeutics 13, 1395–1406 (2013). 24215283 10.1586/14737175.2013.857603

[b15] Rodrigues J. C. . Normal human monocytes exposed to glioma cells acquire myeloid-derived suppressor cell-like properties. Neuro-oncology 12, 351–365 (2010). 20308313 10.1093/neuonc/nop023 PMC2940603

[b16] Raychaudhuri B. . Myeloid-derived suppressor cell accumulation and function in patients with newly diagnosed glioblastoma. Neuro-oncology 13, 591–599 (2011). 21636707 10.1093/neuonc/nor042 PMC3107102

[b17] Finke J. . MDSC as a mechanism of tumor escape from sunitinib mediated anti-angiogenic therapy. International immunopharmacology 11, 856–861 (2011). 21315783 10.1016/j.intimp.2011.01.030 PMC3109226

[b18] Draghiciu O., Nijman H. W., Hoogeboom B. N., Meijerhof T. & Daemen T. Sunitinib depletes myeloid-derived suppressor cells and synergizes with a cancer vaccine to enhance antigen-specific immune responses and tumor eradication. Oncoimmunology 4, e989764 (2015). 25949902 10.4161/2162402X.2014.989764 PMC4404834

[b19] Fujita M. . COX-2 blockade suppresses gliomagenesis by inhibiting myeloid-derived suppressor cells. Cancer research 71, 2664–2674 (2011). 21324923 10.1158/0008-5472.CAN-10-3055 PMC3075086

[b20] Ko J. S., Bukowski R. M. & Fincke J. H. Myeloid-derived suppressor cells: a novel therapeutic target. Current oncology reports 11, 87–93 (2009). 19216839 10.1007/s11912-009-0014-6

[b21] Mills C. D., Kincaid K., Alt J. M., Heilman M. J. & Hill A. M. M-1/M-2 macrophages and the Th1/Th2 paradigm. Journal of immunology 164, 6166–6173 (2000). 10.4049/jimmunol.164.12.6166 10843666

[b22] Hesse M. . Differential regulation of nitric oxide synthase-2 and arginase-1 by type 1/type 2 cytokines *in vivo*: granulomatous pathology is shaped by the pattern of L-arginine metabolism. Journal of immunology 167, 6533–6544 (2001). 10.4049/jimmunol.167.11.6533 11714822

[b23] Condamine T. & Gabrilovich D. I. Molecular mechanisms regulating myeloid-derived suppressor cell differentiation and function. Trends in immunology 32, 19–25 (2011). 21067974 10.1016/j.it.2010.10.002 PMC3053028

[b24] Srivastava M. K., Sinha P., Clements V. K., Rodriguez P. & Ostrand-Rosenberg S. Myeloid-derived suppressor cells inhibit T-cell activation by depleting cystine and cysteine. Cancer research 70, 68–77 (2010). 20028852 10.1158/0008-5472.CAN-09-2587 PMC2805057

[b25] Huang B. . Gr-1+ CD115+ immature myeloid suppressor cells mediate the development of tumor-induced T regulatory cells and T-cell anergy in tumor-bearing host. Cancer research 66, 1123–1131 (2006). 16424049 10.1158/0008-5472.CAN-05-1299

[b26] Youn J. I., Nagaraj S., Collazo M. & Gabrilovich D. I. Subsets of myeloid-derived suppressor cells in tumor-bearing mice. Journal of immunology 181, 5791–5802 (2008). 10.4049/jimmunol.181.8.5791 PMC2575748 18832739

[b27] Highfill S. L. . Bone marrow myeloid-derived suppressor cells (MDSCs) inhibit graft-versus-host disease (GVHD) via an arginase-1-dependent mechanism that is up-regulated by interleukin-13. Blood 116, 5738–5747 (2010). 20807889 10.1182/blood-2010-06-287839 PMC3031417

[b28] Balducci A. . A novel probe for the non-invasive detection of tumor-associated inflammation. Oncoimmunology 2, e23034 (2013). 23526711 10.4161/onci.23034 PMC3601170

[b29] Ardenkjaer-Larsen J. H. . Increase in signal-to-noise ratio of >10,000 times in liquid-state NMR. Proceedings of the National Academy of Sciences of the United States of America 100, 10158–10163 (2003). 12930897 10.1073/pnas.1733835100 PMC193532

[b30] Chaumeil M. M., Najac C. & Ronen S. M. Studies of Metabolism Using (13)C MRS of Hyperpolarized Probes. Methods in enzymology 561, 1–71 (2015). 26358901 10.1016/bs.mie.2015.04.001

[b31] Golman K. . Cardiac metabolism measured noninvasively by hyperpolarized ^13^C MRI. Magnetic resonance in medicine: official journal of the Society of Magnetic Resonance in Medicine/Society of Magnetic Resonance in Medicine 59, 1005–1013 (2008). 10.1002/mrm.21460 18429038

[b32] Keshari K. R. . Hyperpolarized ^13^C dehydroascorbate as an endogenous redox sensor for *in vivo* metabolic imaging. Proceedings of the National Academy of Sciences of the United States of America 108, 18606–18611 (2011). 22042839 10.1073/pnas.1106920108 PMC3219134

[b33] Hu S. . *In vivo* measurement of normal rat intracellular pyruvate and lactate levels after injection of hyperpolarized [1-(13)C]alanine. Magnetic resonance imaging 29, 1035–1040 (2011). 21855243 10.1016/j.mri.2011.07.001 PMC3172390

[b34] Jensen P. R., Karlsson M., Meier S., Duus J. O. & Lerche M. H. Hyperpolarized amino acids for *in vivo* assays of transaminase activity. Chemistry 15, 10010–10012 (2009). 19714690 10.1002/chem.200901042

[b35] Chaumeil M. M. . Non-invasive *in vivo* assessment of IDH1 mutational status in glioma. Nature communications 4, 2429 (2013). 10.1038/ncomms3429 PMC3908661 24019001

[b36] Kurhanewicz J. . Analysis of cancer metabolism by imaging hyperpolarized nuclei: prospects for translation to clinical research. Neoplasia 13, 81–97 (2011). 21403835 10.1593/neo.101102 PMC3033588

[b37] Golman K. & Petersson J. S. Metabolic imaging and other applications of hyperpolarized ^13^C1. Academic radiology 13, 932–942 (2006). 16843845 10.1016/j.acra.2006.06.001

[b38] Chen A. P. . Hyperpolarized C-13 spectroscopic imaging of the TRAMP mouse at 3T-initial experience. Magnetic Resonance in Medicine: official journal of the Society of Magnetic Resonance in Medicine/Society of Magnetic Resonance in Medicine 58, 1099–1106 (2007). 10.1002/mrm.21256 17969006

[b39] Harris T., Eliyahu G., Frydman L. & Degani H. Kinetics of hyperpolarized ^13^C1-pyruvate transport and metabolism in living human breast cancer cells. Proceedings of the National Academy of Sciences of the United States of America 106, 18131–18136 (2009). 19826085 10.1073/pnas.0909049106 PMC2775348

[b40] Park I. . Hyperpolarized ^13^C magnetic resonance metabolic imaging: application to brain tumors. Neuro-oncology 12, 133–144 (2010). 20150380 10.1093/neuonc/nop043 PMC2940577

[b41] Merritt M. E., Harrison C., Storey C., Sherry A. D. & Malloy C. R. Inhibition of carbohydrate oxidation during the first minute of reperfusion after brief ischemia: NMR detection of hyperpolarized ^13^CO_2_ and H^13^CO_3_. Magnetic resonance in medicine: official journal of the Society of Magnetic Resonance in Medicine/Society of Magnetic Resonance in Medicine 60, 1029–1036 (2008). 10.1002/mrm.21760 PMC2696889 18956454

[b42] Schroeder M. A. . *In vivo* assessment of pyruvate dehydrogenase flux in the heart using hyperpolarized carbon-13 magnetic resonance. Proceedings of the National Academy of Sciences of the United States of America 105, 12051–12056 (2008). 18689683 10.1073/pnas.0805953105 PMC2515222

[b43] Laustsen C. . Assessment of early diabetic renal changes with hyperpolarized [1-(13)C]pyruvate. Diabetes/metabolism research and reviews 29, 125–129 (2013). 23166087 10.1002/dmrr.2370

[b44] Lee P. . *In vivo* hyperpolarized carbon-13 magnetic resonance spectroscopy reveals increased pyruvate carboxylase flux in an insulin-resistant mouse model. Hepatology 57, 515–524 (2013). 22911492 10.1002/hep.26028

[b45] MacKenzie J. D. . Detection of inflammatory arthritis by using hyperpolarized ^13^C-pyruvate with MR imaging and spectroscopy. Radiology 259, 414–420 (2011). 21406626 10.1148/radiol.10101921 PMC3079121

[b46] Thind K. . Detection of radiation-induced lung injury using hyperpolarized (13) C magnetic resonance spectroscopy and imaging. Magnetic resonance in medicine: official journal of the Society of Magnetic Resonance in Medicine/Society of Magnetic Resonance in Medicine (2012). 10.1002/mrm.24525 23074042

[b47] Thind K. . Mapping metabolic changes associated with early Radiation Induced Lung Injury post conformal radiotherapy using hyperpolarized (1)(3)C-pyruvate Magnetic Resonance Spectroscopic Imaging. Radiotherapy and oncology: journal of the European Society for Therapeutic Radiology and Oncology 110, 317–322 (2014). 24440041 10.1016/j.radonc.2013.11.016

[b48] Nelson S. J. . Metabolic imaging of patients with prostate cancer using hyperpolarized [1-(1)(3)C]pyruvate. Science translational medicine 5, 198ra108 (2013). 10.1126/scitranslmed.3006070 PMC4201045 23946197

[b49] Shaghaghi H. . Metabolic spectroscopy of inflammation in a bleomycin-induced lung injury model using hyperpolarized 1-(13) C pyruvate. NMR in biomedicine 27, 939–947 (2014). 24865640 10.1002/nbm.3139 PMC4110199

[b50] Keshari K. R. & Wilson D. A. Chemistry and biochemistry of ^13^C hyperpolarized magnetic resonance using dynamic nuclear polarization. Chemical Society Reviews 43, 1627–1659 (2014). 24363044 10.1039/c3cs60124b PMC4086923

[b51] Chiavazza E. . Earth’s magnetic field enabled scalar coupling relaxation of ^13^C nuclei bound to fast-relaxing quadrupolar 14N in amide groups. Journal of magnetic resonance 227, 35–38 (2013). 23262330 10.1016/j.jmr.2012.11.016

[b52] Marigo I. . Tumor-induced tolerance and immune suppression depend on the C/EBPbeta transcription factor. Immunity 32, 790–802 (2010). 20605485 10.1016/j.immuni.2010.05.010

[b53] Hill D. K. . Model free approach to kinetic analysis of real-time hyperpolarized ^13^C magnetic resonance spectroscopy data. PloS one 8, e71996 (2013). 24023724 10.1371/journal.pone.0071996 PMC3762840

[b54] Rodriguez P. C. . Arginase I-producing myeloid-derived suppressor cells in renal cell carcinoma are a subpopulation of activated granulocytes. Cancer research 69, 1553–1560 (2009). 19201693 10.1158/0008-5472.CAN-08-1921 PMC2900845

[b55] Rodriguez P. C. . Arginase I production in the tumor microenvironment by mature myeloid cells inhibits T-cell receptor expression and antigen-specific T-cell responses. Cancer research 64, 5839–5849 (2004). 15313928 10.1158/0008-5472.CAN-04-0465

[b56] London R. E., Walker T. E., Whaley T. W. & Matwiyoff N. A. Nitrogen-15 n.m.r. studies of ^13^C, 15N labeled arginine. Organic magnetic resonance 9, 598–600 (1977).

[b57] Shang H. . Handheld electromagnet carrier for transfer of hyperpolarized carbon-13 samples. Magnetic resonance in medicine: official journal of the Society of Magnetic Resonance in Medicine/Society of Magnetic Resonance in Medicine 75, 917–922 (2016). 10.1002/mrm.25657 PMC4567531 25765516

[b58] Allouche-Arnon H., Lerche M. H., Karlsson M., Lenkinski R. E. & Katz-Brull R. Deuteration of a molecular probe for DNP hyperpolarization–a new approach and validation for choline chloride. Contrast media & molecular imaging 6, 499–506 (2011). 22144028 10.1002/cmmi.452

[b59] Shao A. & Hathcock J. N. Risk assessment for the amino acids taurine, L-glutamine and L-arginine. Regulatory toxicology and pharmacology: RTP 50, 376–399 (2008). 18325648 10.1016/j.yrtph.2008.01.004

[b60] Larson P. E. . Multiband excitation pulses for hyperpolarized ^13^C dynamic chemical-shift imaging. Journal of magnetic resonance 194, 121–127 (2008). 18619875 10.1016/j.jmr.2008.06.010 PMC3739981

[b61] Cheng T. . Automated transfer and injection of hyperpolarized molecules with polarization measurement prior to *in vivo* NMR. NMR in biomedicine 26, 1582–1588 (2013). 23893539 10.1002/nbm.2993

[b62] Ardenkjaer-Larsen J. H. . Dynamic nuclear polarization polarizer for sterile use intent. NMR in biomedicine 24, 927–932 (2011). 21416540 10.1002/nbm.1682

[b63] Batel M. . A multi-sample 94 GHz dissolution dynamic-nuclear-polarization system. Journal of magnetic resonance 214, 166–174 (2012). 22142831 10.1016/j.jmr.2011.11.002

[b64] Park I. . Dynamic hyperpolarized carbon-13 MR metabolic imaging of nonhuman primate brain. Magnetic resonance in medicine: official journal of the Society of Magnetic Resonance in Medicine/Society of Magnetic Resonance in Medicine 71, 19–25 (2014). 10.1002/mrm.25003 PMC4041734 24346964

[b65] Yang W. C., Ma G., Chen S. H. & Pan P. Y. Polarization and reprogramming of myeloid-derived suppressor cells. Journal of molecular cell biology 5, 207–209 (2013). 23532593 10.1093/jmcb/mjt009 PMC3695657

[b66] Khaled Y. S., Ammori B. J. & Elkord E. Myeloid-derived suppressor cells in cancer: recent progress and prospects. Immunology and cell biology 91, 493–502 (2013). 23797066 10.1038/icb.2013.29

[b67] Cherry J. D., Olschowka J. A. & O’Banion M. K. Neuroinflammation and M2 microglia: the good, the bad, and the inflamed. Journal of neuroinflammation 11, 98 (2014). 24889886 10.1186/1742-2094-11-98 PMC4060849

